# Successful Treatment of Severe Tungiasis in Pigs Using a Topical Aerosol Containing Chlorfenvinphos, Dichlorphos and Gentian Violet

**DOI:** 10.1371/journal.pntd.0005056

**Published:** 2016-10-11

**Authors:** Francis Mutebi, Georg von Samson-Himmelstjerna, Hermann Feldmeier, Charles Waiswa, Jeanne Bukeka Muhindo, Jürgen Krücken

**Affiliations:** 1 School of Veterinary Medicine and Animal Resources, College of Veterinary Medicine, Animal Resources and Bio-security, Makerere University, Kampala, Uganda; 2 Institute for Parasitology and Tropical Veterinary Medicine, Freie Universität Berlin, Berlin, Germany; 3 Institute of Microbiology and Hygiene, Charité University Medicine, Berlin, Campus Benjamin Franklin, Berlin, Germany; 4 National Drug Authority, Kampala, Uganda; Hitit University, Faculty of Medicine, TURKEY

## Abstract

**Background:**

In endemic communities, zoonotic tungiasis, a severe skin disease caused by penetrating female sand fleas, is a public health hazard causing significant human and animal morbidity. No validated drugs are currently available for treatment of animal tungiasis. Due to the reservoir in domestic animals, integrated management of human and animal tungiasis is required to avert its negative effects.

**Methods and principal findings:**

A topical aerosol containing chlorfenvinphos 4.8%, dichlorphos 0.75% and gentian violet 0.145% licensed to treat tick infestations, myiasis and wound sepsis in animals in the study area, was tested for its potential tungicidal effects in a randomized controlled field trial against pig tungiasis in rural Uganda. Animals with at least one embedded flea were randomized in a treatment (n = 29) and a control (n = 26) group. One week after treatment, 58.6% of the treated pigs did not show any viable flea lesion whereas all control pigs had at least one viable lesion. After treatment the number of viable lesions (treated median = 0, overall range = 0–18 vs. control median = 11.5, range = 1–180) and the severity score for estimating acute pathology in pig tungiasis (treated median = 1, range = 0–3.5 vs. control median = 7, range = 0–25) were significantly lower in treated than in control pigs (p < 0.001). In the treatment group the median number of viable flea lesions decreased from 8.5 to 0 (p < 0.001). Similarly, the median acute severity score dropped from 6 to 1 (p < 0.001). Every pig in the treatment group showed a decrease in the number of viable fleas and tungiasis-associated acute morbidity while medians for both increased in the control group.

**Conclusions:**

The study demonstrates that a topical treatment based on chlorfenvinphos, dichlorphos and gentian violet is highly effective against pig tungiasis. Due to its simplicity, the new approach can be used for the treatment of individual animals as well as in mass campaigns.

## Author Summary

Infection with the sand flea *Tunga penetrans* causes severe disease in humans and animals. There are no validated drugs for treatment of animal tungiasis preventing implementation of integrated tungiasis control interventions targeting human and animal infections. A field trial was conducted to evaluate the effects of a commercial insecticidal aerosol containing chlorfenvinphos 4.8%, dichlorphos 0.75%, and gentian violet 0.145%, which is licensed to treat tick infestations, myiasis and wound sepsis, on tungiasis in pigs, the major animal reservoir in rural hyperendemic villages in Uganda. Infected pigs were recruited and randomly assigned to treatment (n = 29) and control (n = 26) groups. Seven days after a single application of the aerosol onto the affected body parts, almost 60% of the treated pigs were cured while all control pigs had at least one penetrated sand flea. The number of viable sand fleas and the severity of the tungiasis were significantly lower in the treated pigs than in the controls. This demonstrates for the first time that a simple and effective topical treatment for animal tungiasis based on two organophosphate insecticides and an antibacterial agent can be used to cure individual animals and can be integrated in tungiasis control campaigns.

## Introduction

Tungiasis is a parasitosis of humans and animals caused by embedded female sand fleas belonging to the genus *Tunga*. So far 14 different *Tunga* species have been described [1–4] of which three have been reported in domestic animals: *Tunga hexalobulata*, *Tunga trimamillata* and *Tunga penetrans* [3, 5]. Only the latter two species are known to be zoonotic. Zoonotic tungiasis is a neglected tropical disease [6, 7] endemic in sub-Saharan Africa and Latin America. The disease is common among the poorest of the poor [3]. In endemic areas, tungiasis is characterized by seasonal fluctuations as well as a high prevalence and intensity in marginalized communities [8–15]. Sometimes, infections in humans and companion animals are also documented in non-endemic areas due to travelers coming from endemic areas [16–22].

With a size of about 1 mm in length, the sand flea is the smallest known member of the order Siphonaptera. After penetration, the female flea undergoes neosomy to attain a size of up to 13 mm in diameter in about two weeks. It remains viable for four to six weeks after which it dies *in situ* [23, 24]. While in the host skin, the burrowed flea provokes severe inflammation which in the majority of cases is complicated by secondary bacterial infections [25, 26]. The resultant morbidity is characterized by a wide range of manifestations and sequelae, some of which are potentially life-threatening [27–29].

Animal infections often occur alongside human infections but the main animal reservoir hosts vary between endemic areas [3, 30–33]. While dogs, cats and rodents have been reported as major animal hosts of sand fleas in South America [30, 31], pigs and, to some extent, dogs were described as important animal reservoirs in Africa [32, 33]. However, there are only a few studies describing the role of animals in the epidemiology of tungiasis and it might well be that there are additional important species depending on socio-cultural and environmental conditions in the particular areas. Animal tungiasis is believed to amplify prevalence and infection intensity of human tungiasis and vice versa [31, 33]. The importance of an animal reservoir depends on the number of sand fleas it harbors since the number of eggs expelled is directly proportional to the infection intensity. Moreover, the degree of overlap between animal ranges and human activities is important since the mobility of fleas is not very high. Therefore, animals kept in or close to human houses can contaminate the immediate environment of the inhabitants with eggs that develop to larvae, pupae and finally host-seeking imagos.

In Bugiri District, Busoga, south eastern Uganda where the study was conducted, pigs were identified as the major animal reservoir for tungiasis and under poor husbandry they may carry several hundred embedded sand fleas during the peak of the transmission season [33]. This does not only cause severe disease in these animals [34] but also predisposes humans to infections through the shedding of eggs around or even in human dwellings [33].

Approaches aiming to achieve control of tungiasis in both, humans and domestic animals, are required for the efficient control of tungiasis in endemic communities [35]. In addition to proper animal husbandry practices, which are often difficult to implement in impoverished villages with very poor infrastructure, treatment protocols for infected animals would be highly beneficial. However, there is no approved drug treatment for animal tungiasis [36, 37]. Studies aimed at improving this situation are very scarce. A recent case report on goat tungiasis suggests that a topical spray containing chlorfenvinphos 4.8%, dichlorphos 0.75% and gentian violet 0.145% (Supona aerosol, previously Pfizer, now Zoetis, South Africa) has tungicidal properties [38]. The objective of this study was to systematically evaluate potential curative effects of the Supona aerosol on pig tungiasis. Supona aerosol is licensed to prevent wound infections and for treatment against ticks and blowfly maggots in cattle, sheep, goats, horses and dogs. The present study shows that a single treatment of pigs with Supona aerosol significantly reduced the intensity and severity of pig tungiasis within seven days.

## Materials and Methods

### Study area and population

The study was carried out in three parishes (Makoma, Wakawaka and Bulidha) of Bugiri District, Busoga, south eastern Uganda. Initially, pigs, which are the most important animal hosts of *T*. *penetrans* in the area [33], were examined. in ten villages These villages were purposively selected because they had a high prevalence of human and pig tungiasis [33]. All pigs in the villages were under a “back yard management system” whereby they were tethered under trees close to human houses but occasionally released to roam freely in search for food. Supplementation was occasionally done with household refuse and plant residues. Pigs which met the inclusion criteria were drawn from four villages, i.e. Busakira, Busindha and Masolya in Makoma Parish and Kibuye located in Wakawaka Parish [33]. The study villages are situated near each other and are characterized by the same physical and socio-economic characteristics. A detailed description of the study area was presented previously [33].

### Study design

The study had a non-blinded, parallel design with one treatment and one control group. For treatment, Supona aerosol containing chlorfenvinphos 4.8%, dichlorphos 0.75% and gentian violet 0.145% was sprayed on the affected body parts. The spray was applied once and the efficacy of the treatment was assessed after seven days. All examinations were carried out by the same investigator (F.M.). The study was conducted between February 8, 2015 and March 17, 2015 which is the peak of the dry season when the attack rate of sand fleas is assumed to be high [39]. During the entire study period, the weather conditions remained unchanged (dry season with no rain). All pig-rearing households in ten villages were identified with the help of the Chairpersons of Local Council One, i.e. the political heads of the villages, or their representatives. After explaining the study objectives, an informed written consent was obtained from the pig owners and a census of pigs in the household was conducted. The feet of all pigs in the households were cleaned with water and a brush and examined systematically as described before [33]. Infected pigs were marked with ear tags and their biographic information was obtained by interviewing the pig owners since there were no records kept by the farmers. During pig examination, sand flea lesions (embedded sand fleas, mutilations of embedded fleas and scars remaining after death of the flea) were counted and staged according to the Fortaleza classification [23]. Clinical manifestations of tungiasis were described in detail for each topographic body area and recorded on individual forms. Photographs of pathological alterations were taken and their respective serial numbers were recorded for later review.

Clinical diagnosis of tungiasis was based on the features of the various stages as described before [23, 24]. Briefly, a dark spot of about 1–2 mm usually in the middle of a hyperemic area (stage II) or a round glassy to yellow often raised patch with a central dark spot of about 4–13 mm in diameter (stage III) characterize the viable stages. Viability was also assessed by recognized viability signs, namely expulsion of eggs, excretion of feces and release of a watery secretion on palpation of the lesions. Dead fleas present as circular and raised brown to black patches surrounded by necrotic tissue (stage IV) while an extruded sand flea is represented by an epidermal circular shallow crater with necrotic edges (stage V). Fresh sores with or without sand flea chitin remnants at the predilection sites were considered as mutilated sand flea lesions. The sand fleas collected from pigs in the study area were identified previously to be *T*. *penetrans* [33].

Only pigs with at least one viable sand flea (stage II-IIIa) and which were not expected to leave the study area for the next three weeks after the baseline examination were included in the study. Pigs meeting these inclusion criteria were randomly allocated in each household into two groups (control or treatment). Randomization at household level was chosen to exclude households as a confounding factor. A paper-based lottery system was used for the random allocation of treatments. If only one pig was present in the household, two papers reading 'treatment' and 'control' were placed in a bowel and one was drawn. If two or more pigs were present, ear tag numbers were drawn. If an uneven number of animals was included from the same household, an additional blank paper was included. In households with five pigs or more and a highly variable intensity of *T*. *penetrans* infection, pigs were stratified into two groups depending on the intensity of infection (i.e. light infection ≤ 5 lesions and heavy infection intensity > 5 lesions) before randomization. This procedure was chosen to exclude the possibility that heavily infected animals would by chance be allocated to the same group.

### Description of treatment details

The pigs in the treatment group were treated once topically with Supona aerosol by covering all the digits (with or without embedded sand fleas) up to the coronary band at the metacarpal and metatarsal joints of the respective limbs (S1 Fig). For lesions occurring at other topographic areas, the aerosol was only applied to sites with embedded sand fleas. No intervention was undertaken for the pigs in the control group until the end of the study period (day 7) after which they were also treated with Supona aerosol. Pig owners were requested to watch for any side effects among pigs after treatment, especially signs of acute toxicosis and anaphylaxis which were explained to them, such as salivation, diarrhea, tremors, convulsions and collapse and to report them to the investigator as soon as detected. In case a pig could not be traced at the time of the visit, the household was revisited again on the same day or one day later.

### Evaluation of treatment effects

The primary outcome measure was the change in the number of viable embedded *T*. *penetrans*. In addition, the severity score for acute pig tungiasis (SSAPT) which was modified from a similar score for human tungiasis [40] and changes in this score were considered as a secondary outcome measure. Pigs were followed up only once, seven days after enrolment and treatment. The outcome measures were assessed at the individual animal level. For this purpose, the following parameters were determined: the number of viable sand fleas of the different stages, the number of morphological/topographic sites (in particular individual digits but for ectopic lesions also other body parts as detailed below for determination of the morbidity score) with viable sand fleas, the total number of sand fleas (viable plus already dead) and the SSAPT obtained as described below. The SSAPT allows the semi-quantitative assessment of morbidity using the clinical pathological features of tungiasis. Since acute morbidity correlates with the number of embedded sand fleas and decreases with reduction of the infection intensity, the SSAPT can be reliably used to quantify the severity of morbidity in a fairly objective manner [41–46]. In heavily infected pigs with mutilated lesions (S2 Fig) it was difficult to count the number of dead fleas and differentiating the mutilated lesions from those that had died naturally. In such cases only lesions which were distinct were analyzed.

The clinical variables used to quantify the SSAPT of the infected pigs were edema, hyperemia, ulcers, fissures, clusters of lesions, mutilation of lesions (as an indicator of itching), pain on digital pressure, ectopy of lesions, suppuration and/or abscessation and alteration of pig gait. The clinical features outlined above either involve a single or a cluster of lesions (e.g. local hyperemia) or affect the entire defined area (digit) or change the general behavior of the animal (e.g. limping). The digits were used as the primary units for the scoring of the morbidity because the majority of the lesions localized here. The pig distal limb was divided into four topographic sites constituted by each of the principal and accessory digits up to the distal metacarpal or metatarsal joints, hence a total of 16 digits for the four limbs of a pig. All other areas were considered ectopic sites of sand flea attachment. For signs that could be localized to each of the digits irrespective of extent, a score of one to three was assigned depending on the number of sites involved (Table 1). For other clinical manifestations, scores ranging from one to three were allocated depending on the relative significance of the sign on the wellbeing of the pig as proposed before [40]. For ectopic localizations, a score of 0.5 was allocated for every ectopic site up to a maximum of eight ectopic sites. Therefore, the maximum score (SSAPT) for an individual pig would be 27 (23+4). How the SSAPT is composed is depicted in Table 1.

**Table 1:** Severity score for acute pig tungiasis (SSAPT).

Characteristic clinical signs	Number of topographic sites affected	Score assigned
Hyperemia and/or edema^a^	1–5	1
	6–10	2
	11–16	3
Pain on digital pressure	1–5	1
	6–10	2
	11–16	3
Suppuration and/or abscess formation^a^	1–5	1
	6–10	2
	11–16	3
Clustering of lesions^b^	1–5	1
	6–10	2
	11–16	3
Fissure (s)^a^	1–5	1
	6–10	2
	11–16	3
Skin ulcerations^a^	1–5	1
	6–10	2
	11–16	3
Mutilation of lesions irrespective of sites involved^c^		2
Altered gait/lameness		3
Ectopy of lesions		0.5^d^

^a^Irrespective of number of foci and size of the area involved on a designated topographical site

^b^Three or more lesions in close proximity (1–2 mm apart).

^c^Mutilation of lesion reflects intense itching

^d^For each ectopic discrete body part involved up to a maximum of eight ectopic sites; maximum 4 points

### Ethics statement

The animal study adhered to the “Animals (Prevention of Cruelty) act”, chapter 39, Constitution of Uganda and the National Drug Policy and Authority (Conduct of Ectoparasiticides Field Trials) Regulations, 2014. Ethical approval of the study was given by the ethical committee of the College of Veterinary Medicine, Animal Resources and Biosecurity, Makerere University (Ref. VAB/REC/14/101), the National Drug Authority (Ref. 462/NDA/DID/02/2016) and the National Council of Science and Technology (Ref. HS 1621). Written consent was obtained from pig owners. After the study all infected pigs in both groups which were still available were treated with Supona aerosol until all lesions were cleared.

### Statistical analysis

Data was entered into Excel (Microsoft Office, 2007), double checked for errors which might have occurred during data entry and transferred to Stata Software package, Version 13 (Stata Corporation, College Station, Texas 77845 USA) and R 3.1.2 software for analysis. Descriptive statistics were generated. The PropCIs package in R was used to calculate 95% confidence intervals (CI) as Wilson score intervals. The binomial test was used to determine if frequencies of mutually exclusive outcomes were significantly different from 50% and the mid p exact test (as implemented in the epitools 0.5–7 package in R) was used to compare proportions between two groups. Since variables were not normally distributed, medians and range of values (minimum to maximum) are mainly provided. The Mann-Whitney U test was used to compare numerical data between groups while the Wilcoxon Matched Pairs Signed Rank Test was used to compare paired data from the same individual animal before and after treatment. Only pigs that were available at day 0 and day 7 after treatment were included in the statistical analyses. Spearman correlations coefficients were calculated with the cor.test command while logistic regression analysis was conducted using the glm function implemented in R software.

Generalized linear mixed models for numbers of viable embedded sand fleas (count data) and ordinal SSAPT data were obtained using a Bayesian approach as implemented in the MCMCglmm 2.22.1 R package. The MCMCglmm command was conducted to run a Monte-Carlo-Markov-Chain with 2 million iterations to estimate model parameters and a Poisson model or an ordinal model were chosen for flea numbers and SSAPT, respectively. All other parameters in MCMCglmm were not changed from default values. Group (control vs. treatment) and time point (before vs. after treatment) were used as two levels of categorical variables and the animal number as random effect. Villages, sex and age of the pigs were considered as potential confounding independent variables. Household was not considered as an independent variable since randomization was performed at the household level and the number of households was relatively high (n = 39) compared to the number of pigs (n = 55). Variables were eliminated stepwise in order to improve the Deviance Information Criterion (DIC).

Effect sizes were calculated using the non-parametric Cliff’s delta using a consistent estimation of the variance approach [47] with the function cliff.delta in the R package effsize 0.6.2. For this purpose, numbers of viable fleas or SSAPT values were compared (i) within the treatment group before and after treatment and (ii) between control and treatment group on day 7 post treatment.

Risk ratios describing the “risk to improve” (lower number of viable fleas or lower SSAPT) were calculated using the risk ratio function in the epitools R package with 95% CI determined by bootstrapping with 5000 iterations. A mid-p exact test was used to determine if the risk ratio was significantly different from 1.

## Results

### Description of the study groups

Out of 497 pigs examined in 141 households, 62 (12.5%) pigs met the inclusion criteria and were enrolled into the study. These pigs belonged to 23 households (16.3%). Overall, a median of 2 pigs and range of 1–6 pigs met the inclusion criteria per household. Of the enrolled pigs, 32 were randomly allocated to the treatment group and 30 to the control group. In the treatment group, three pigs were not available at follow up, and in the control group four pigs were lost. This limited the number of pigs included in the data analysis to 29 and 26, respectively. The number of pigs recruited per group did not differ significantly (p = 0.788 in a binomial test). Animal recruitment and randomization into groups is depicted in the flow diagram in S3 Fig. Table 2 summarizes the demographic characteristics of the two groups of pigs that were finally included in the analysis. Pigs in the treatment group were slightly older than those in the control group (p = 0.282 in a Man-Whitney U test). While the number of male pigs did not differ from that of the female pigs in the control group (p = 0.845), the treatment group had a higher number of females than males (p = 0.024 in a binomial test).

**Table 2:** Demographic characteristics of the study groups of pigs included in the trial

Characteristic	Treatment group	Control group	p value
Total number of pigs recruited	29	26	0.788^c^
Number of households^a^	20	18	0.871^c^
Number of pigs included per household: median (range)	2 (1–3)	2 (1–3)	0.993^d^
Village^b^			
Busindha	4	1	0.375^c^
Masolya	9	7	0.804^c^
Kibuye	11	12	1.000^c^
Busakira	5	6	1.000^c^
**Breed**	All mixed breeds	All mixed breeds	
**Sex**			
Male	8	12	0.845^c^
Female	21	14	0.024^c^
Age in months: Median (range, minimum to maximum)	8 (2–24)	6 (2–18)	0.282^d^

^a^To which the enrolled pigs belonged"

^b^To which the households with enrolled pigs belonged

^c^In a binomial test

^d^In a Mann-Whitney U test.

All pigs had lesions on the digits of the legs but six pigs also had lesions on ectopic sites such as skin along the metacarpal and metatarsals (n = 6), snout (n = 2), tail (n = 1) and testes (n = 1). Many lesions on the digits were mutilated and appeared either as fresh sores with chitin sand flea remnants or as necrotic hollow black ghosts. Viable lesions were mostly detected at the edges of the mutilated sites (S4 Fig).These were often accompanied by adjacent skin abrasions and/or ulcers at the predilection sites. Infected pigs had lesions at up to 21 distinct topographic sites.

### Treatment effects

**Fig 1:**
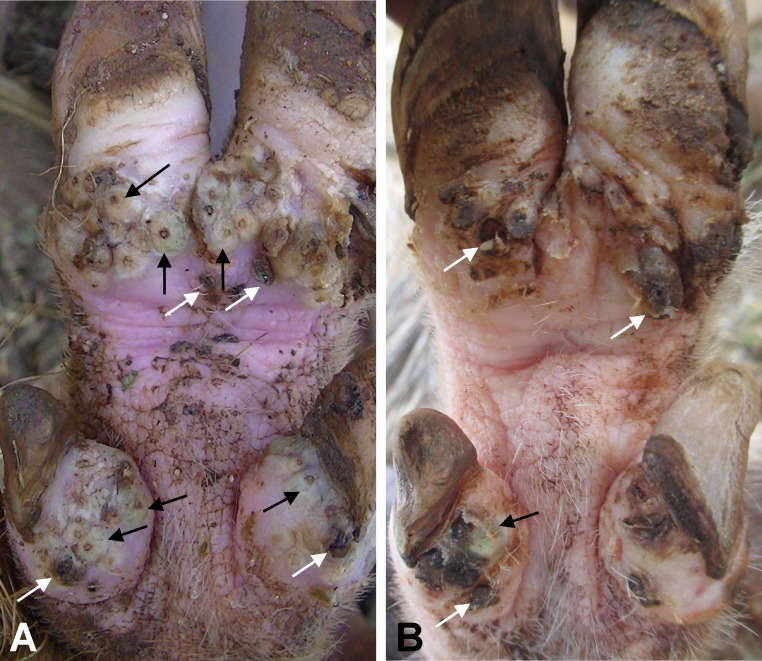
**Left hind leg digits of a pig before (A) and after (B) treatment.** This pig had 297 lesions before treatment (208 viable and 89 dead) of which 6 were ectopic on metacarpal and metatarsal skin. Only three viable lesions were detectable after treatment. Selected viable lesions are marked with black arrows while some dead lesions are marked with white arrows.

**Fig 2:**
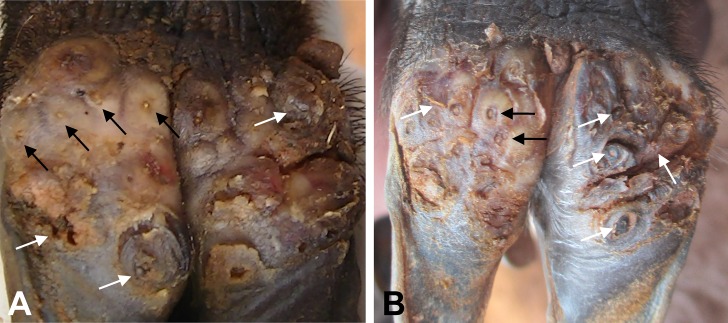
**Digits of a pig in the control group at baseline (A) and after seven days (B).** This pig had 29 lesions of which 21 were viable (black arrows) and eight were dead at baseline but one week later there were 17 viable lesions and more than 28 dead lesions many of which were mutilated (white arrows).

**Fig 3:**
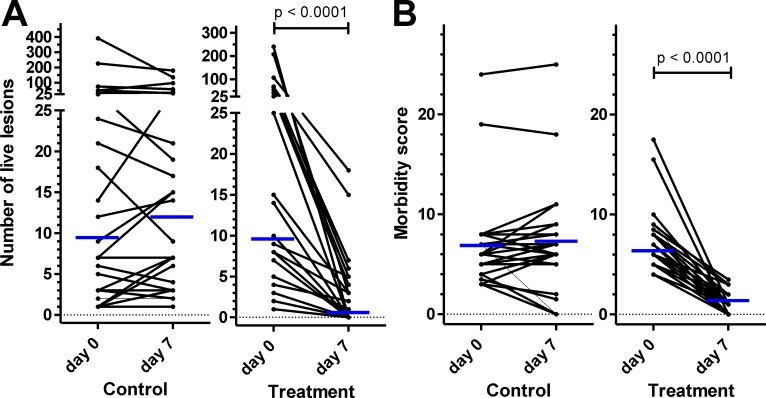
**Number of viable lesions (A) and morbidity scores (B) in control and treatment groups of pigs at base line and 7 days treatment.** Data belonging to the same animal in day 0 and day 7 are connected by a line. Medians are shown as blue lines.

No adverse effects were reported by the owners in any of the treated pigs and no pigs were excluded due to such effects. Before treatment, the number of viable fleas was high in both groups with a median of 8 (range 1–392) for the control group and a median of 9 (range 1–241) for the treatment group. The median SSAPT was 6.5 (range 3–24) in the control and 6 (range 4–17.5) in the treatment group. Following treatment, the number of viable and dead lesions dropped significantly (Table 3). The later was presumably attributed to exteriorization of the lesions (Fig 1 and S5 Fig). On day 7 after treatment, 58.6% (95% CI 40.74–74.49%) of the pigs in the treatment group did not show any viable lesion and were considered to be cured. The highest number of viable lesions recorded in the treatment group at day 7 was 18. This pig had 241 viable sand fleas before treatment. In contrast, all pigs in the control group had at least one viable lesion at day 7 (cure rate = 0%; 95% CI 0–12.87%) and one pig had up to 180 viable lesions (Table 3). Differences between the treatment group on day 7 and (i) the untreated control on day 7 and (ii) the treatment group at day 0 were highly significant (p < 0.001 in a Mann-Whitney-U test and a Wilcoxon Matched Paired Signed Rank test). Because of massive mutilation of lesions among the untreated pigs by scratching the affected sites (Fig 2 and S6 Fig), the number of viable lesions slightly decreased in some pigs but overall, there was an increment in the number of viable lesions and in the SSAPT in the control group (Fig 3). In contrast, there was a highly significant reduction in the number of viable penetrated fleas and in the SSAPT for the treatment group. In fact, both values decreased for every individual pig after treatment (Fig 3).

**Table 3:** Comparison of clinical characteristics of the two groups of pigs at baseline and after intervention.

	Control group (n = 26)		Treatment group (n = 29)		P-values			
Variable	Summary statistics		Summary statistics		control vs. treatment^a^		Day 0 vs. day 7^b^	
	Day 0	Day 7	Day 0	Day 7	Day 0	Day 7	Control	Tratment
Number of animals with manipulated lesions (%)	25 (96.15%)	23 (88.5%)	29 (100%)	0 (0%)	0.473	p<0.001	0.360	<0.001
Median number of sites with viable lesions (range)	4.5 (1–20)	6 (1–20)	6 (1–21)	0 (0–7)	0.445	p<0.001	0.447	<0.001
Median number of lesions per animal (range)	14 (1–832)	21.5 (1–838)	18 (1–756)	9 (0–120)	0.367	0.046	0.038	<0.001
Median number of viable lesions (range)	8 (1–392)	11.5 (1–180)	9 (1–241)	0 (0–18)	0.473	p<0.001	0.807	<0.001
Stage II: median (range)	0 (0–4)	0 (1–11)	0 (0–19)	0 (0–4)	0.463	0.125	0.832	0.273
Stage IIIa: median (range)	8 (1–392)	9.5 (1–177)	8 (1–241)	0 (0–14)	0.543	p<0.001	0.931	<0.001
Stage IIIb: median (range)	0 (0–17)	0 (0–11)	0 (0–8)	0 (0 (0–6)	0.826	0.078	0.63	0.14
Median number of dead lesions per animal (range)	6.5 (0–440)	9.5 (0–702)	10 (0–515)	6 (0–102)	0.348	0.715	0.006	0.516
Median acute morbidity score (range)	6 (3–24)	7 (0–25)	6 (1–17.5)	1 (0–3.5)	0.574	p<0.001	0.536	<0.001

^a^Calculated using the mid-p exact test for percentages and the unpaired Mann-Whitney U test for comparison of medians between groups at the same time-point.

^b^Calculated using the mid-p exact test for percentages and the Wilcoxon Matched Pairs Signed Rank Test for comparison of medians between groups at the same time-point.

Using logistic regression, no significant effect of the number of viable fleas at baseline on complete clearance was detectable (p = 0.224). However, the median number of viable fleas at baseline was 7.5 (range 1–241) in those animals that had completely cleared the infection on day 7 after treatment but was 34.5 (range 4–208) in the animals that still had at least one viable flea. This difference was statistically significant (p = 0.012 in a Mann-Whitney U test). The animal with the highest number of viable fleas before treatment (241) was completely cured by treatment.

Overall, there was a high correlation between the number of viable flea lesions and the SSAPT (Spearman’s rho = 0.731, p<0.001) indicating that severity of disease increased with numbers of embedded fleas and that both parameters are well suited to determine potential effects of treatment.

### Regression analyses

In order to determine the effects of potential confounding variables, the paired datasets (two groups × two time points) were analyzed using generalized linear mixed models. The considered independent variables age, sex and village consistently dropped out of all models since they always increased the DIC.

For numbers of viable fleas (count data), the best model with the lowest DIC included the variables group and time point and their interaction (Table 4). Only the interaction between treatment and time point on day 7 after treatment had a significant effect on numbers of embedded sand fleas. The average reduction in the number of viable fleas was approximately 15 (e^2.697^) in the treatment group on day 7 compared to the reference categories control group and day 0. This difference was highly significant (p<0.001).

**Table 4:** Generalized linear mixed regression model for changes in number of viable fleas calculated using a Monte-Carlo-Markov-Chain (MCMC) approach.

Coefficient	Posterior mean	95% Confidence interval	P value (MCMC)
Intercept	2.293	1.726–2.845	<0.001
Day 7^a^	0.106	-0.262–0.478	0.570
Treatment group^a^	0.142	-0.628–0.924	0.718
Day 7*Treatment group	-2.697	-3.232–-2.082	<0.001
DIC	619.6		

^a^Reference categories are day 0 and the control group.

With regard to the ordinal SSAPT data, a model containing only the variables group and time point was again clearly preferred according to the DIC. This model also showed that significant effects were only observed in the combination of treatment with the time point day 7 after treatment resulting in an average decrease in the SSAPT of 1.44 in this combination (Table 5).

**Table 5:** Generalized linear mixed regression model for changes in the severity score for acute pig tungiasis (SSAPT) calculated using a Monte-Carlo-Markov-Chain (MCMC) approach.

Coefficient	Posterior mean	95% Confidence interval	P value (MCMC)
Intercept	0.884	0.616–1.438	<0.001
Day 7^a^	0.176	-0.396–0.498	0.524
Treatment group^a^	-0.056	-0.537–0.260	0.871
Day 7*Treatment group	-1.441	-2.100–-0.538	<0.001
DIC	636.2		

^a^Reference categories are day 0 and the control group.

### Effect sizes

Effect sizes for treatment with Supona aerosol were calculated non-parametrically as Cliff’s delta values. For the number of viable fleas, a comparison in the treatment group before and after treatment provided a Cliff’s delta of 0.76 (95% CI: 0.53–0.89) and comparison of control and treatment group on day 7 had a similar value of 0.76 (95% CI: 0.55–0.88). For the SSAPT, the comparison of values for the treatment group before and after treatment led to a Cliff’s delta of 0.97 (95% CI: 0.93–0.98) while the comparison between control and treatment group was somewhat lower with a Cliff’s delta of 0.79 (95% CI: 0.54–0.91). Since all Cliff’s delta values ≥0.474 are generally considered as “large” effects [48], all calculations indicate that the effects of a single Supona aerosol treatment on the number of viable fleas or the morbidity score are not only significant but also large.

In order to determine how different the probabilities of pigs in the two groups were to improve (decreased number of viable fleas or decreased SSAPT) between both visits, risk ratios were calculated. In the treatment group, the risk ratio to have a decreased number of viable fleas compared to the control group was 2.36 (95% CI: 1.63–4.33; p<0.001) in a mid-p exact test). Similarly, the risk ratio to show a reduced SSAPT in the comparison between treatment and control group was 2.89 (95% CI: 1.86–6.50, p<0.001).

## Discussion

The present study shows for the first time curative efficacy of a drug against pig tungiasis in a randomized, controlled trial using previously validated outcome measures, namely viability of embedded sand fleas and an acute morbidity score. Such an approach has never been used in animal tungiasis. The results clearly show that treatment with the Supona aerosol significantly reduced the number of viable embedded sand fleas and tungiasis-associated morbidity. The large effects sizes as determined by Cliff’s delta for both, number of viable fleas and SSAPT, clearly show that the treatment had not only a statistically significant effect but considerably improved the health of the pigs. The risk ratios above 2 furthermore show that improvement was observed much more often in the treatment group than in the control group. In fact, every single treated animal had a decrease in the number of viable fleas and in the SSAPT.

The duration of the study was limited by the end of the dry season but a second visit after 14 days would have been possible for many animals included in the study. However, this was not done since there was a considerable problem with compliance of farmers owning pigs in the control group. Seeing that the health status of the animals in the treatment group had improved so much, they claimed that the animals in the control group should also be treated. For this reason and due to considerations regarding animal welfare, all animals were treated at the second visit.

To date, only a few drugs have been tested to treat animal tungiasis with variable outcomes. The most important classes of ectoparasiticides on the market, macrocyclic lactones and pyrethroids, would be considered obvious candidates to treat animal tungiasis. In particular, the endectocide ivermectin, which would also cure several other frequently co-endemic parasitoses such as scabies, ascariosis, human hookworm disease as well as pig infections with strongylid gastrointestinal nematodes, is an obvious candidate to be evaluated for its effects against *T*. *penetrans*. Indeed, ivermectin has been used several times to treat tungiasis.

A single case report of dog tungiasis from Denmark suggested that ivermectin is an effective drug [49]. However, the dog was treated subcutaneously with ivermectin, which is not licensed for treatment of dogs due to severe adverse effects in mdr-1 deficient animals. It is most important to note that the authors reported only that lesions resolved within one month, a time span when most lesions would have resolved without treatment anyhow. A randomized controlled trial found only minimal effects of oral ivermectin treatment on embedded sand fleas in humans [50].

In a case serious on pigs in Tanzania, Cooper applied lindane (hexachlorocyclohexane), an organochloride on *T*. *penetrans* lesions. The author claimed that lindane was effective but no data was presented to support the statement [51, 52]. Topical applications of an oily solution of Trichlorphone 0.2% (Neguvon) applied on embedded sand fleas in dog foot pads was also reported to be tungicidal [53]. Again, no convincing data was provided. In another attempt to treat tungiasis in pets, neither Neguvon nor a collar containing a combination of propoxur and flumethrin (Kiltix) were successful [35]. A variety of other drugs such as niridazole [54], albendazole, thiabendazole [55], trichlorphone and potassium permanganate [37, 43] have been used in humans to treat tungiasis but all these were proven to have negligible effects on embedded sand fleas.

Another field trial reported a reduction in the number of sand fleas in dogs following application of a spot on in the neck with a formulation containing 10% imidacloprid and 50% permethrin (Advantix) [56]. The study was not conceived as a randomized control trial. The reduction of the number of embedded sand fleas was very slow and no data were provided on the viability of the embedded sand fleas. It seems that only new infections were prevented, presumably due to repellent and not due to insecticidal effects of the product and the already embedded sand fleas persisted and died *in situ* at the end of their normal life span. Alternatively, the slow onset of the drug effect might be caused by a slow distribution from the neck, where the drug was applied, to the footpad, where sand fleas are embedded. Moreover, the number of lesions re-increased already three weeks after treatment indicating that the drug did not protect against newly invading *T*. *penetrans* for four weeks as it does against other flea species such as the cat flea *Ctenocephalides felis* [57].

In the current field trial, the drug was applied directly on the lesions. This obviously achieved a local concentration of the active ingredient lethal to the embedded sand fleas. The dual action of the formulation–killing the embedded sand fleas and acting as an antimicrobial agent–presumably contributed to the quick reduction of inflammation, thus facilitating the shedding of dead fleas and repair of tissue damage. This explains the rapid reduction in the severity score for acute tungiasis of the treated pigs. The persistence of a few viable lesions following treatment may have resulted from failure of the chemical reaching the embedded sand fleas, e.g. due to the necrotic debris on the skin. Indeed, the median number of embedded viable fleas before treatment was significantly higher in those pigs that still had at least one viable flea after treatment. However, the number of viable fleas was not a significant variable in the logistic regression analysis suggesting that additional parameters influence the effects of the drugs.

It has been demonstrated before in humans that the intensity of infection correlates strongly with the clinical severity of tungiasis [39]. Measures of acute morbidity (SSAPT) dramatically decreased with a reduced intensity of infection, whereas measures of chronic morbidity only slightly change over time, since the latter reflect the severity of previous tungiasis episodes [45]. Therefore, changes in acute morbidity scores are an appropriate outcome measure to determine the efficacy of interventions against tungiasis. Since Supona aerosol treatment strongly decreased the number of viable lesions and concomitantly ameliorated acute morbidity, conclusions of the study are strongly supported by the data despite the lack of precision regarding the number of lesions in some highly infected animals.

The results of the present study suffer from three major limitations: (i) the difficulties to count the number of all lesions and viable lesions in clusters, (ii) the loss of initially viable lesions due to mutilation by the pigs and (iii) the short duration of the study. Clustering of lesions is a common feature of animal and human tungiais [25, 34]. Pruritus of lesions is often associated with extensive mutilation of lesions, and abrasions or ulcerations of the skin, resulting from intense rubbing of affected sites against objects. Mutilations of lesions and clustering of lesions are inevitable problems in a randomized controlled trial which limit the evaluation of individual lesions. Severity of tungiasis appears to increase with the size of the neosome, the infection intensity and the associated clustering of lesions [25]. In severe cases, it is therefore likely that the number of lesions is underestimated which can also lead to underestimation of the effects of the drug. Although only semi-quantitative, the acute morbidity score is a useful alternative to the number of viable lesions to determine the effects of drug treatment. While all the treated pigs did not show any evidence of itching or pain as indicated by absence of new skin abrasions or lesion excoriations, almost all untreated pigs had persistent or newly mutilated lesions, abrasions or skin ulcerations at the end of the study.

Regarding the third limitation, the short duration of the follow-up period, a longer follow up period as well as inclusion of a third group with multiple treatments compared to a groups with a single treatment would have been desirable. However, this study was a follow up to a larger study [33] and there were no extramural funds available to perform additional field work. Moreover, as mentioned above, pig owners were usually not willing to leave their pigs untreatment throughout the study period. Thus, analysis of long-term effects and determination of an optimized treatment protocol with a recommended application frequency to maximize protective effects while minimizing the costs for the farmers is a subject of future studies.

In Uganda, Supona aerosol is commonly used as a wound antiseptic in animals, and it is applied to prevent wound sepsis, myiasis and tick infestations. Chlorfenvinphos and dichlorphos are organophosphates that are used as acaricides and insecticides with cholinesterase inhibitory effects while gentian violet is a triarylmethane compound with antifungal, antibacterial as well as anthelminthic properties. A combination of antibacterial and insecticidal properties in addition to the ease of application appear to make the Supona aerosol a good choice for treatment of tungiasis in animals since secondary infections with bacteria are common in tungiasis. Effects of control measures in domestic animals can be expected to reduce the burden of human tungiasis as well hence animal treatment should be considered at least in outbreak situations when money for control is available from public health and governmental institutions. Quantitative studies comparing human and animal tungiasis between intervention and control villages are required to demonstrate such effects. Chemical control of animal tungiasis should be integrated with other control practices such as public health education, animal confinement, hygienic housing and treatment of humans.

A complete blinding during the study was not possible since it would have required different people doing assessment/treatment and evaluation of effects but there were not enough personnel to do that. Initially, it was intended to conduct the study with blinding of the examiner in a way that he would not have access to the records from the intervention day during the follow-up visit. However, the differences in flea numbers and in particular in morbidity were so obvious that is was immediately clear to which group the animal belonged. Of course, the missing blinding might have introduced bias into the study. However, the quantitative differences between both groups were so large that the general conclusions can be considered to be valid.

## Conclusions

The study has demonstrated a therapy using a simple, effective and rapidly acting topical drug against severe tungiasis in livestock. The combined antibacterial effects of gentian violet and the ectoparasitic effects of chlorfenvinphos plus dichlorphos make Supona aerosol a prototype formulation for treatment of animal tungiasis. The formulation can be used to treat individual cases and has the potential to be used in systematic treatment of infected animals to eliminate *T*. *penetrans* animal reservoirs in the endemic communities with minimal technical and financial resource inputs. As such these findings are of major importance for future One Health based tungiasis control initiatives which are urgently needed in the endemic regions.

## Supporting Information

S1 Fig Pig digits with several sand flea lesions covered with Supona aerosol. All digits of pigs in the treatment group, irrespectively of whether they had sand fleas or not, were covered with the spray (purple coloration).
(PDF)

S2 Fig Pig digits with several sand fleas some of which are mutilated by the animal (black arrows). (PDF)

S3 Fig Flow diagram describing animal recruitment, allocation into groups and losses during the study according to the guidelines of the CONSORT statement (http://www.consort-statement.org/). (PDF)

S4 Fig Heavy infection and extensive mutilation of sand flea lesions on all digits with viable (arrows) sand fleas evident at the edges of the mutilated and necrotic areas. (PDF)

S5 Fig **Digits of the left front leg before (A) and after treatment (B).** This pig had 180 viable (black arrows) and another 396 dead lesions (white arrows) before treatment. On day 7 after treatment, only seven viable embedded sand fleas were detected.
(PDF)

S6 Fig **Right front leg of a pig in the control group at baseline (A) and 7 days later (B).** At baseline, this pig had 49 viable and 35 dead lesions but a week later it had a total of 181 discernible lesions of which 98 were viable (black arrows) while 83 were mutilated (white arrows).
(PDF)
