# Supplementary figures and images for: Successful Treatment of Severe Tungiasis in Pigs Using a Topical Aerosol Containing Chlorfenvinphos, Dichlorphos and Gentian Violet
Source: PLoS Negl Trop Dis. 2016 Oct 11;10(10):e0005056. doi: 10.1371/journal.pntd.0005056 (PMC5058476; doi:10.1371/journal.pntd.0005056)

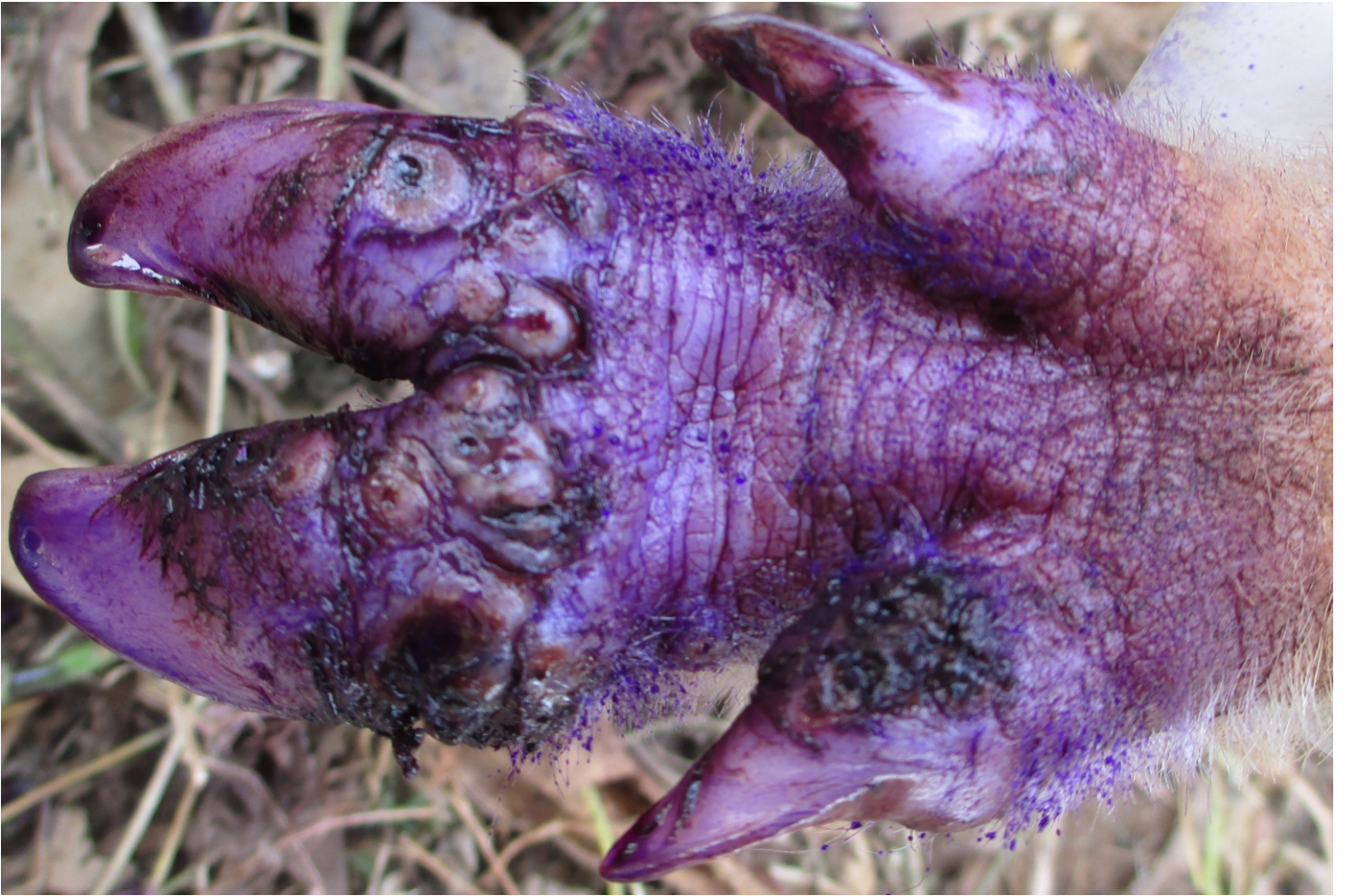

Supplement: S1 Fig — All digits of pigs in the treatment group, irrespectively of whether they had sand fleas or not, were covered with the spray (purple coloration). (PDF) [file pntd.0005056.s001.pdf]

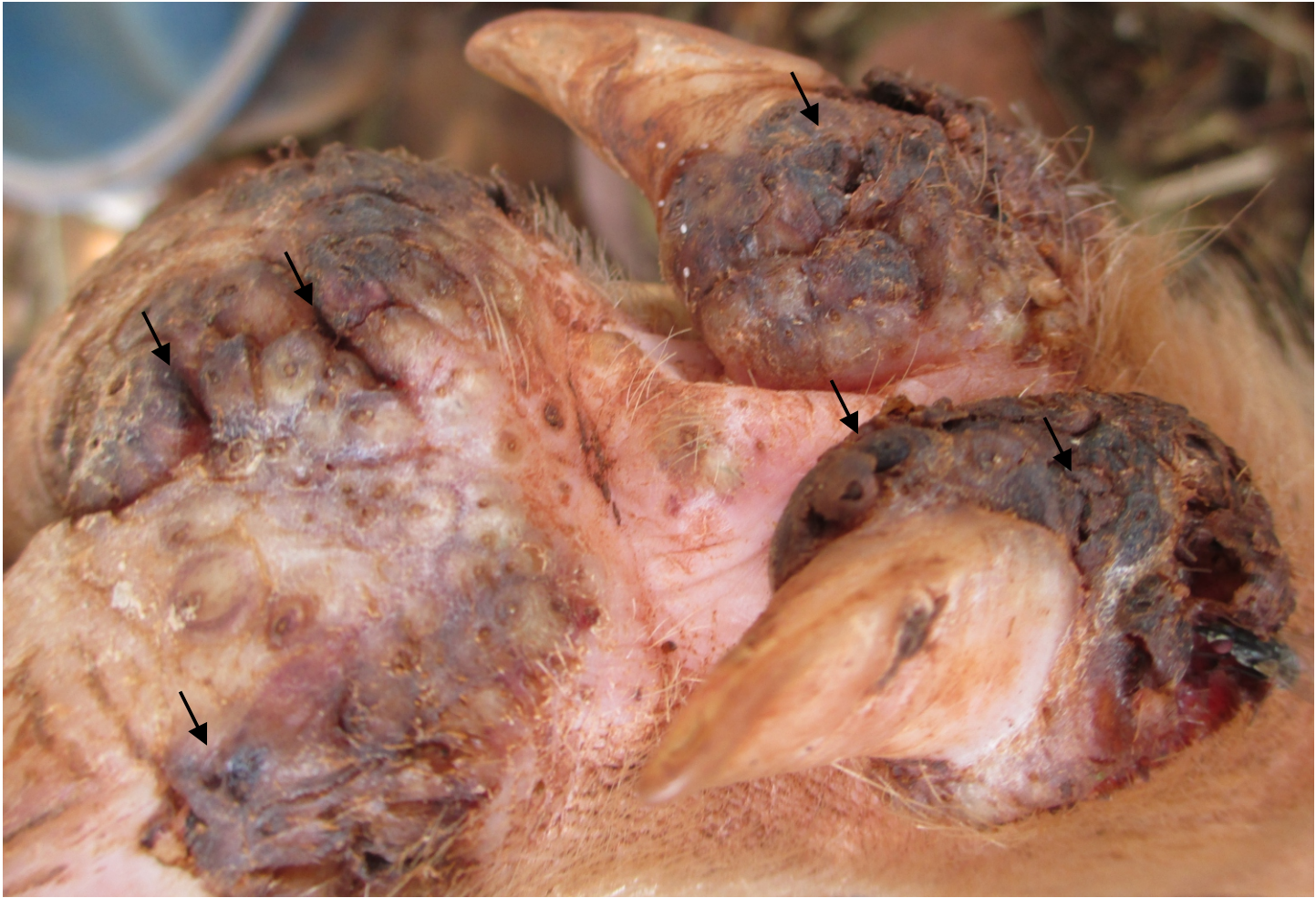

Supplement: S2 Fig — (PDF) [file pntd.0005056.s002.pdf]

**S3 Fig: Flow Diagram showing recruitment and randomization of the pigs to the groups**

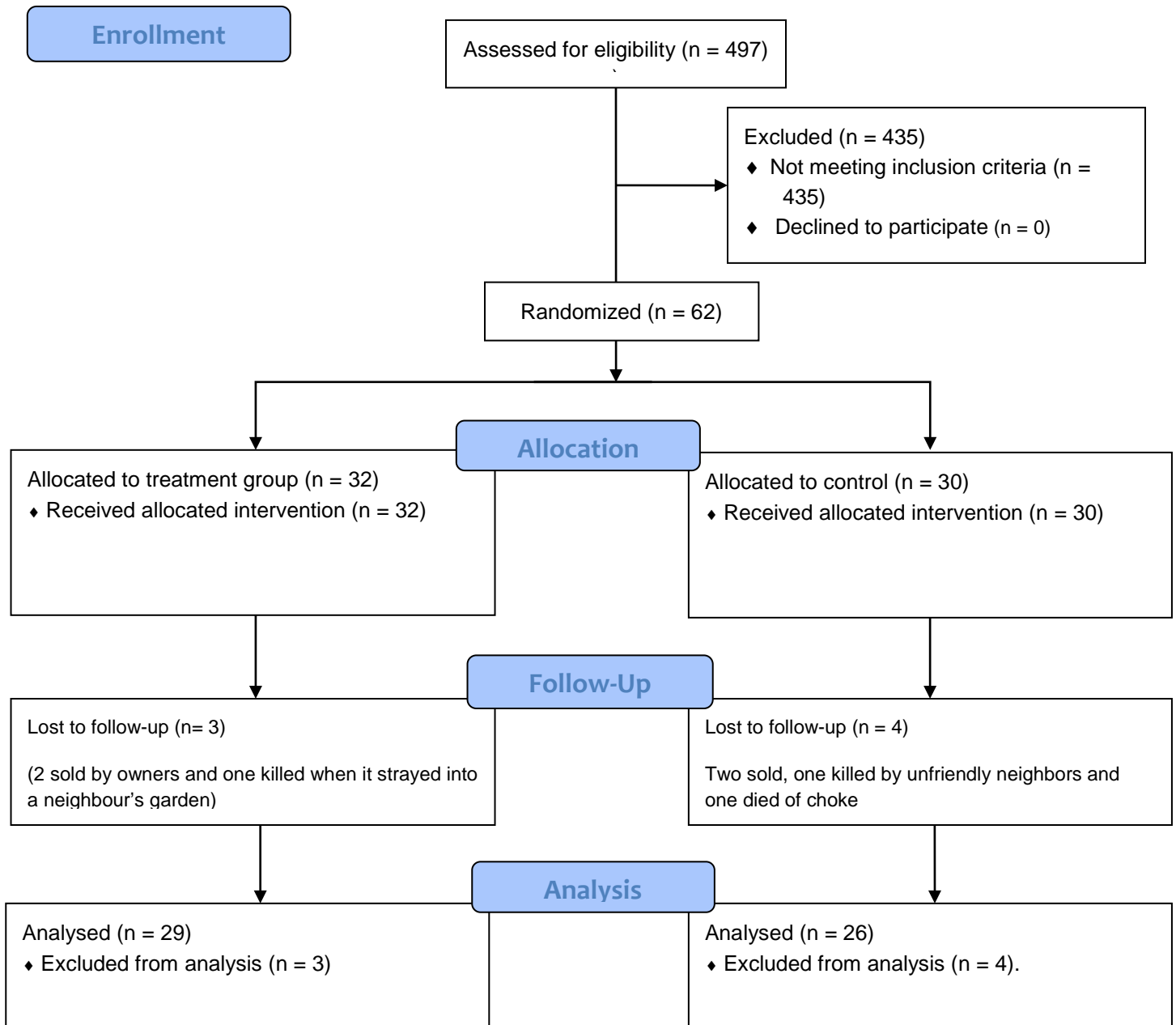

Supplement: S3 Fig — (PDF) [file pntd.0005056.s003.pdf]

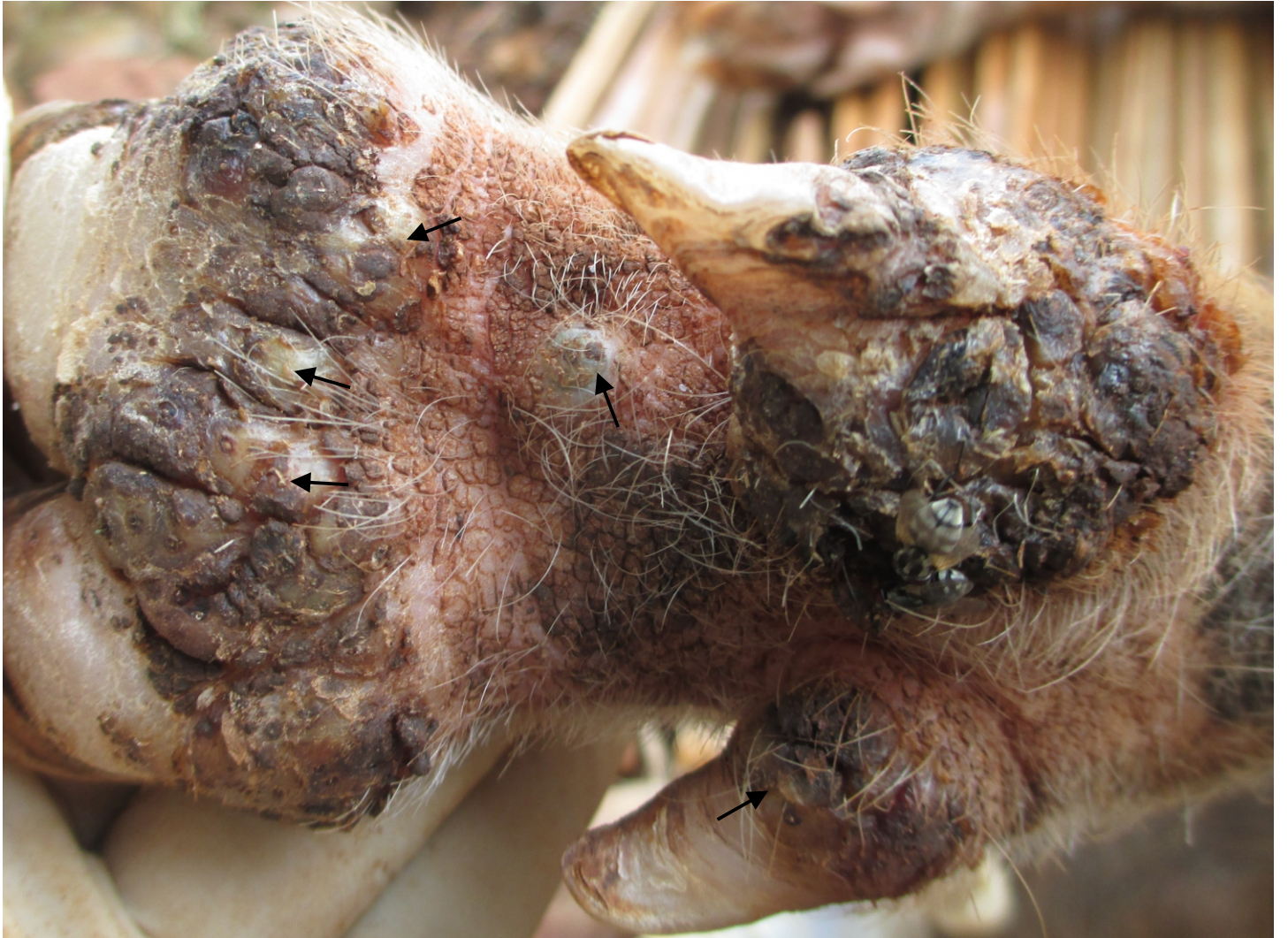

Supplement: S4 Fig — (PDF) [file pntd.0005056.s004.pdf]

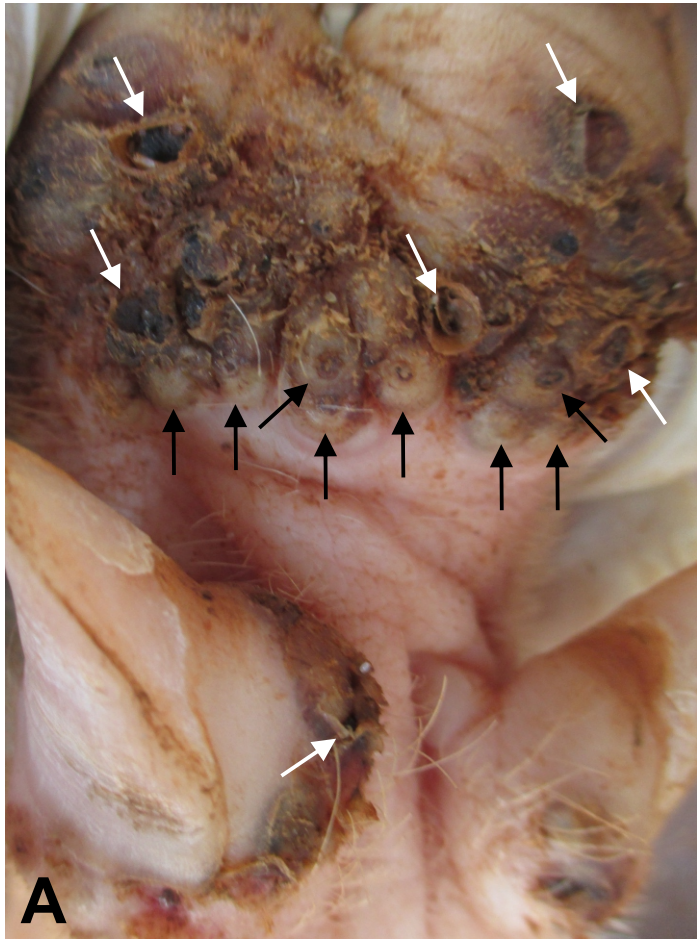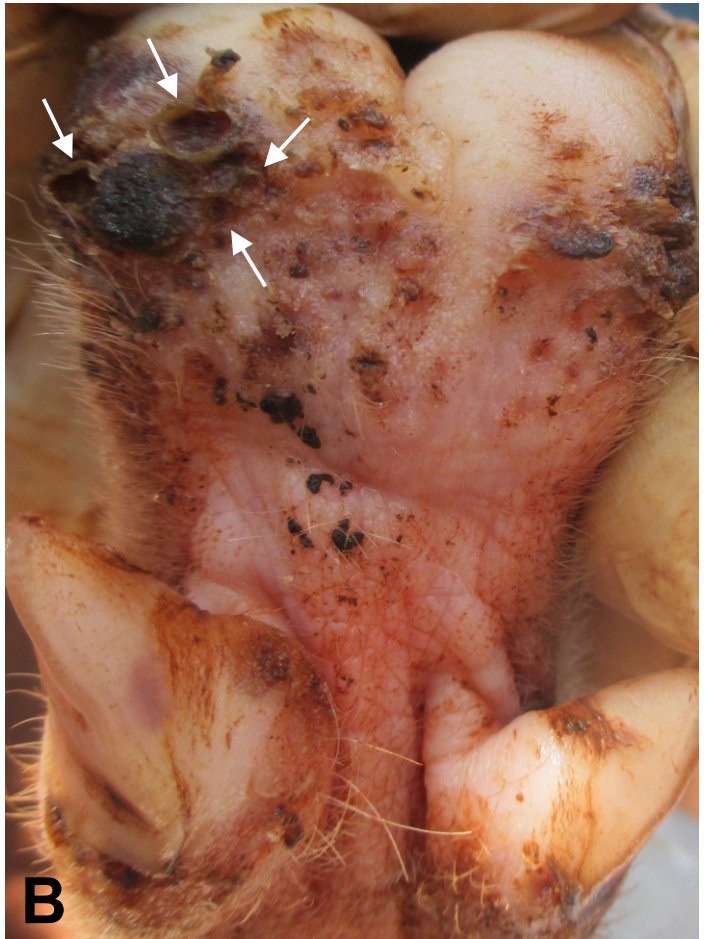

Supplement: S5 Fig — Digits of the left front leg before (A) and after treatment (B). This pig had 180 viable (black arrows) and another 396 dead lesions (white arrows) before treatment. On day 7 after treatment, only seven viable embedded sand fleas were detected. (PDF) [file pntd.0005056.s005.pdf]

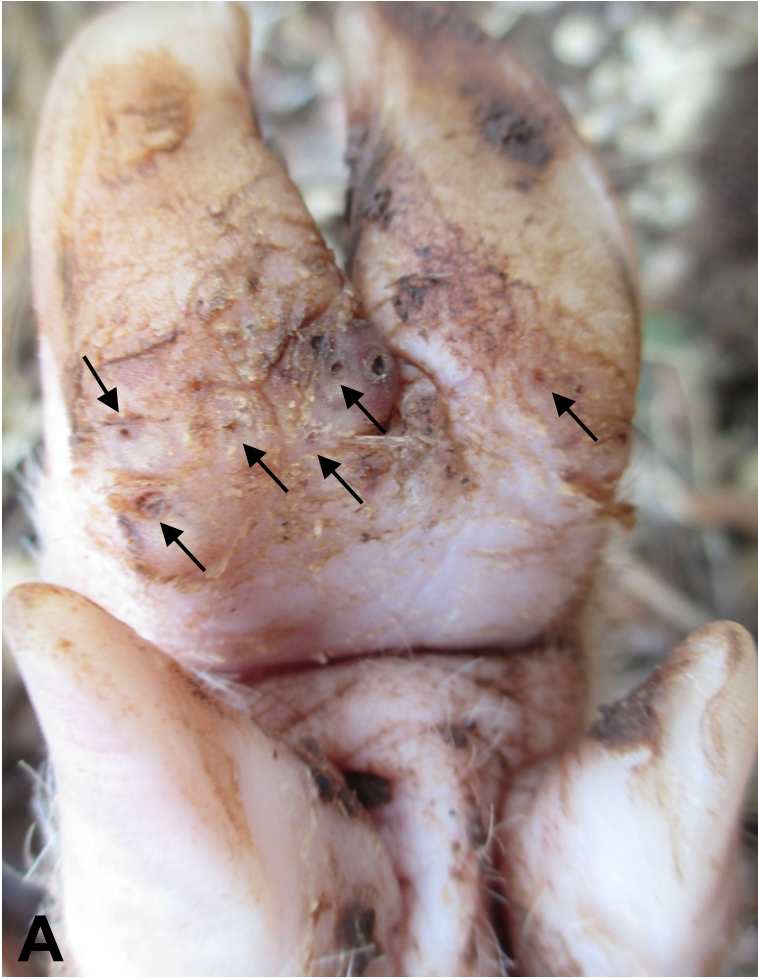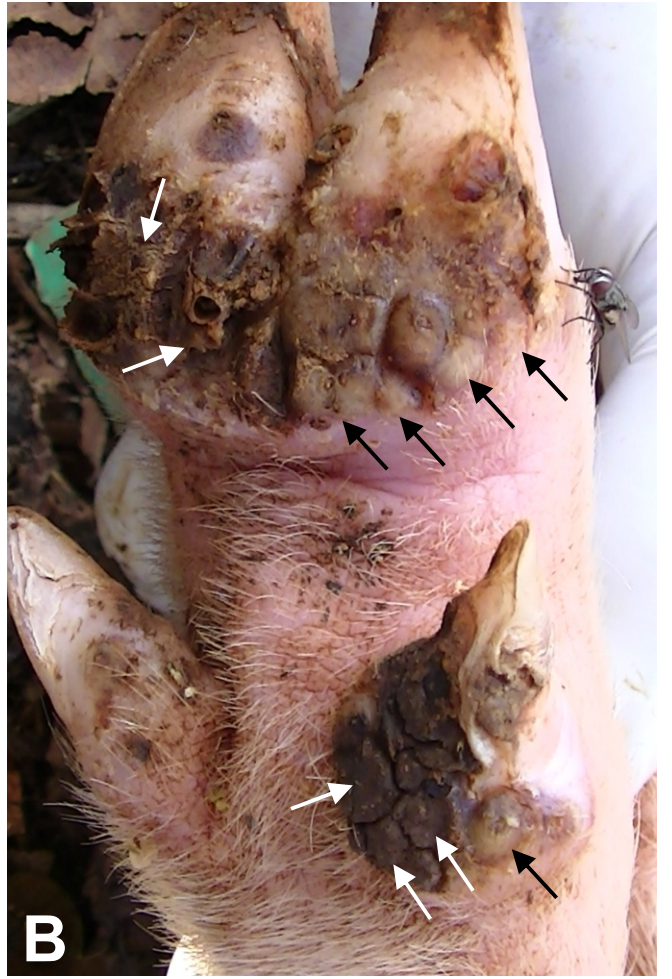

Supplement: S6 Fig — Right front leg of a pig in the control group at baseline (A) and 7 days later (B). At baseline, this pig had 49 viable and 35 dead lesions but a week later it had a total of 181 discernible lesions of which 98 were viable (black arrows) while 83 were mutilated (white arrows). (PDF) [file pntd.0005056.s006.pdf]
